# County Medical Societies

**Published:** 1888-04

**Authors:** 


COUNTY MEDICAL SOCIETIES.—Counry Medical Societies or associations composed of the physicians from two or three counties, which meet monthly or quarterly, are more beneficial to the practitioner than the State Associations which meets only once a year. Dr. A. N. Carrigan, in a paper read before the Arkansas Medical Association, very truthfully remarks upon this subject that, "Too many medical men think too little or too lightly of their county societies, forgetting the fact that it is the very fountain of power and greatness. No man arrives at distinction or honor in our state and national councils who has not served his time in some county or municipal society. It is the school for the young physician to learn in, and it is only a matter of time for him to arrive at local distinction if he will carefully study and report to his county medical society all of his cases of interest. It stimulates him to study and enables him to become accurate in diagnosis and successful in treatment. Mind needs the friction of opposing mind if it ever grows and develops into anything more than it was when it started Too many of our young physicians are like young wasps, as big when they are hatched as they ever get to b› afterwards. This state of affairs is a reproach to the well qualified young men that come into our State every year graduated from first-class medical schools. Do these young men drop into the Sleepy-Hollow ways of the ancient Arkansas doctor ? If they do not, where is the evidence to the contrary ? Have we any increase in the number of County Medical Societies ? With a few honorable exceptions we have no evidence of progress in scientific work, even in the old societies, much less of the formation of new ones or of their work. How much of this state of affairs is chargeable to the older members of the profession I will not pretend to say, but one thing we all know is certain, and that is, if we do not bring the people by whom we are surrounded up to our standard, we will in turn drop down to theirs."

While all this is true, our State Associations are highly important and should not be neglected. The older members of the prefession should encourage and stimulate the younger, and the younger members should honor and respect the older, that the meetings may be both pleasant and profitable, that they may carry home with them agreeable and lasting recollections of each othei, and more exalted views of the profession and of the objects which called them together.

THE HOMILETIC REVIEW for March is up to the high-water line which the previous numbers of the year reached. "Harmful Books," by Prof. T. W. Hunt, of Princeton College, is an able and discriminating paper well worthy of careful reading. Dr. Armstrong, of Virginia, discusses "The Christian Evidences" in the light of recent criticism, with great clearness and force. Dr. Sprecker's paper, "Huxley on Miracles," is noteworthy as showing the famous scientist's concessions of the truth. "Was Adam the First Man?" by Dr. C. S. Robinson, will startle many by its array of Scripture and suggestive queries. The Sermonic Section has two full and able sermons by Dr. Lyman Abbott and Dr. G. Lansing Taylor, and six outlines by Drs. Kittredge, E. H. Johnson, E. McChesney, Wayland Hoyt, and Wray and Heath of England. Two admirable exegetical articles by Rev. C. M. Corben, Ph.D., and Dr. Howard Crosby, are, as usual, fully sustained in point of interest, variety and helpful material to the preacher, pastor, and Christian worker.

Published by Funk & Wagnalls, 18 and 20 Astor Place, New York. $3.00 per year ; 30 cents per single number.

IN THE MARCH *Magazine of American History* there is a most agreeable variety of entertaining and scholarly papers. The freshness and timeliness of themes considered in each issue of this priceless periodical are phenomenal. Among the many interesting papers we mention "Historic Cannon Balls and Houses ;" "New York and Ohio Centennial," by Douglas Campbell ; Mrs. Ole Bull on "Lief Erikson ;" "Central Ohio Seventy Years Ago ;" "Escape of Grant and Meade" from riding into the enemy's lines in 1864 ; "With Cortez in Mexico, 1519." As usual, the number is a specimen of typographic beauty, unexcelled in the magazine field. Price, $5 a year. 743 Broadway, New York City.

